# Lovastatin overcomes gefitinib resistance through TNF-α signaling in human cholangiocarcinomas with different LKB1 statuses *in vitro* and *in vivo*

**DOI:** 10.18632/oncotarget.4408

**Published:** 2015-06-23

**Authors:** Sheng-Huei Yang, Hung-Yun Lin, Vincent H.S. Chang, Chien-Chung Chen, Yun-Ru Liu, Jinghan Wang, Keqiang Zhang, Xiaoqing Jiang, Yun Yen

**Affiliations:** ^1^ PhD Program for Cancer Biology and Drug Discovery, College of Medical Science and Technology, Taipei Medical University, Taipei, Taiwan; ^2^ Taipei Cancer Center, Taipei Medical University, Taipei, Taiwan; ^3^ Institute of Translational Medicine, Taipei Medical University, Taipei, Taiwan; ^4^ Graduate Institute of Biomedical Materials and Engineering, Taipei Medical University, Taipei, Taiwan; ^5^ Office of Human Research, Taipei Medical University, Taipei, Taiwan; ^6^ The First Department of Biliary Surgery, Eastern Hepatobiliary Surgical Hospital, The Second Military Medical University, Shanghai, China; ^7^ Department of Molecular Pharmacology, City of Hope National Medical Center and Beckman Research Center, Duarte, California, USA

**Keywords:** cholangiocarcinomas, combination therapy, lovastatin, gefitinib

## Abstract

Gefitinib resistance has been shown to complicate cancer therapy. Lovastatin is a proteasome inhibitor that enhances gefitinib-induced antiproliferation in non-small cell lung cancer. The objective of this study is to investigate the mechanism of lovastatin-induced antiproliferation in gefitinib-resistant human cholangiocarcinoma.

Two gefitinib-resistant cholangiocarcinoma cell lines, SSP-25 and HuH-28, were used in this study to determine how to compensate gefitinib resistance. The combined effect of these two drugs was examined using the MTT assay, qPCR, immunoblotting, flow cytometry, and *in vivo* xenograft. Results indicated that lovastatin enhanced TNF-α-induced cell death *in vitro*. In addition, the combination of lovastatin with gefitinib enhanced accumulation of TNF-α. Furthermore, the treatment induced a synergistic cytotoxic effect and antiproliferation through apoptosis in SSP-25 cells and cell cycle arrest in HuH-28 cells. Reproductive results were also observed in *in vivo* xenografts. These observations suggest that the combination of gefitinib and lovastatin might have additive antiproliferative effects against gefitinib-resistant cholangiocarcinoma cells. Based on these observations, we concluded that the combination of gefitinib and lovastatin could be used to overcome gefitinib resistance in cholangiocarcinoma cells.

## INTRODUCTION

Cholangiocarcinomas are malignant tumors of the intra- or extrahepatic biliary tract and are the second most common type of primary liver cancer. An increasing incidence of cholangiocarcinoma has been documented. They are associated with a high rate of mortality because of the difficulty in early detection and their resistance to chemotherapeutic agents. 15% of the EGFR gene mutations in the kinase domain are in biliary cancer [1]. K-ras mutation and aberrant expression of p53 are present in one-third of the intrahepatic cholangiocarcinomas [2]. These mutations lead to the resistance to many chemotherapeutic agents in cholangiocarcinomas [3, 4] but there is no standard therapy in this situation [5, 6].

LKB1 (also known as serine-threonine kinase 11, STK11) is a tumor suppressor, which is mutated in various cancers including cholangiocarcinomas [7]. Physiologically, LKB1 possesses multiple cellular functions in the regulation of cell cycle arrest, cell bioenergetics metabolism, embryo development, cell polarity, and apoptosis [8]. LKB1 has been shown to regulate PAK-LIMK-cofilin and cyclin D1/CDK4 pathways [9]. The overexpression of the LKB1 protein significantly inhibits tumor growth in breast cancer cells [10]. The phosphorylation of LKB1 is regulated by several growth factors including EGF and TNF-α [7]. Mutation or loss of LKB1 drives tumorigenesis in several types of tumors [11–14].

Epidermal growth factor receptor (EGFR) has been reported to play an important role in pathogenesis in biliary tract carcinoma [15]. Activation of EGFR is sustained following EGF stimulation in cholangiocarcinoma cells. The prolonged EGFR activation results in extended ERK1/2 activation in cholangiocarcinoma cells [16]. K-ras belongs to a group of genes that encode a family of guanosine triphosphate (GTP)-binding proteins involved in regulating prosurvival signaling pathways downstream from EGFR [17]. Mutant K-ras renders the constitutively GTP-bound protein, eventually resulting in the activation of the MEK-ERK and the PI3K-AKT antiapoptotic pathways. AKT activation is associated with disease progression in lung adenocarcinoma with wild-type EGFR in patients treated with gefitinib. Administering IGF1R-TKI decreases AKT signaling and restores gefitinib sensitivity in mutant K-ras cells [18]. Gefitinib has been reported to be a radiosensitiser, which inhibits radiation-induced phosphorylation of EGFR and the downstream pathway and therefore enhances radiosensitivity in cholangiocarcinoma cells [1, 19].

Lovastatin is a 3-hydroxy-3-methylglutaryl coenzyme A (HMG-CoA) reductase inhibitor; its inhibitory action on HMG-CoA reductase leads to the depletion of isoprenoids, which inhibits posttranslational modification of K-ras [20]. The effect of the combination of lovastatin with gefitinib on gefitinib-resistant human nonsmall-cell lung cancer (NSCLC) A549 and NCI-H460 cell lines with K-ras mutations has shown to effectively downregulate K-ras protein and suppress the Raf, ERK1/2, AKT, and EGFR phosphorylation in both cell lines [20]. These effects are correlated with both the upregulation of cleaved caspase-3, poly (ADP-ribose) polymerase (PARP) and Bax, and the downregulation of Bcl-2. Similar results are observed in the combined treatment of lovastatin (1 μM) and gefitinib inhibits proliferation, which promotes cell apoptosis, and reduces the AKT activity in K-ras mutant NSCLC cells compared with gefitinib alone [21].

In this study, two cholangiocarcinoma SSP-25 cells and HuH-28 cells were treated with gefitinib, lovastatin, or their combination. Conversely, both gefitinib and lovastatin induced cell cycle arrest through LKB1 in HuH-28 cells. On the other hand, gefitinib induced autophagy and lovastatin induced apoptosis in SSP-25 cells. Furthermore, inducible TNF-α by lovastatin played one of cruicial roles in lovastatin-induced antiproliferation. The addition of gefitinib did enhance lovastatin-induced TNF-α expression and thereafter antiproliferation occurred. The observations were reproduced in xenograft modeling. These studies indicated that the combined treatment of gefitinib and lovastatin induced a synergistic cytotoxic effect through increased TNF-α expression and caused antiproliferation in cholangiocarcinomas with different LKB-1 statuses.

## RESULTS

### The combined treatment of gefitinib and lovastatin induced antiproliferation in human intrahepatic cholangiocarcinoma SSP-25 and HuH-28 cells

**Figure 1:**
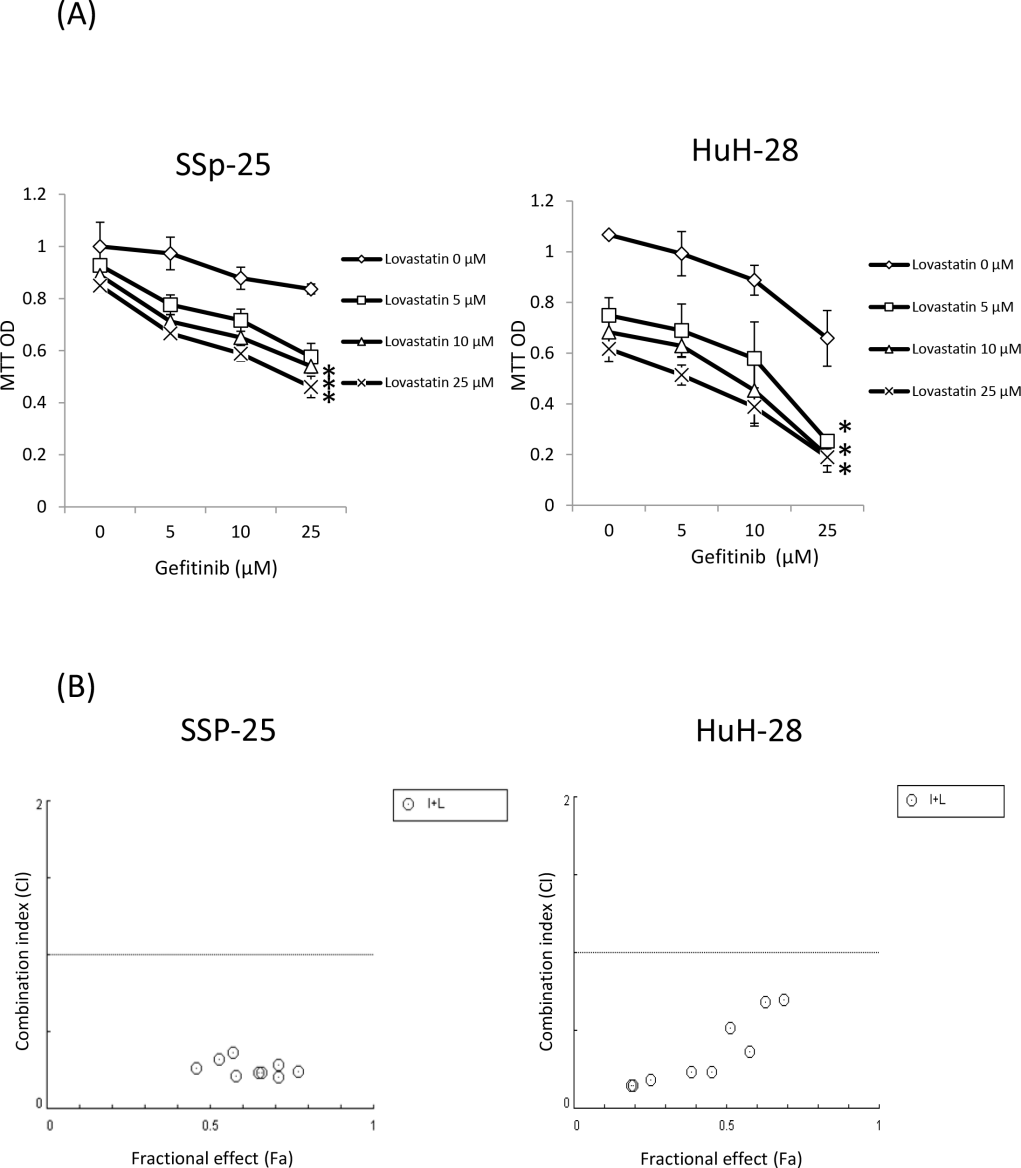
Combined treatment of lovastatin and gefitinib induced antiproliferation in human intrahepatic cholangiocarcinoma SSP-25 and HuH-28 cells **A.** SSP-25 and HuH-28 cells grown in 96-well trays were treated with lovastatin (L), gefitinib (G), or their combination (G + L) for 72 hours. Cell viability was detected using the MTT assay. **B.** Combination index (CI) values were calculated using the cell survival values in CompuSyn software, as described in Materials and Methods. Student's *t* test was conducted and considered significant at *p* < 0.05 (*)

The effect of the combination of gefitinib and lovastatin to attenuate the viability of SSP-25 and HuH-28 cells was evaluated using the MTT assay. As shown in Fig. 1A, SSP-25 and HuH-28 cells were resistant to gefitinib; however, the combined treatment of gefitinib and lovastatin significantly decreased viability in a concentration-dependent manner. For combined treatments, a fixed concentration ratio, also determined using the cell survival values, was applied. CI values were calculated using the CompuSyn software, as described in Materials and Methods; the representative Fa-CI plots are shown in Fig. 1B. The combined ratio of 1:1 was with lower combination index and with ED:50 8.34 μM and 8.748 μM in SSP-25 and HuH-28 cell lines. The fixed concentration of gefinitib/lovastatin:10 μM/10 μM was used in the study. The effect of gefinitib, lovastatin and the combination was also examined in YSCCC, a cholangiocarcinoma cell line which is more sensitive to gefitinib. The similar addative effect in the combined treatment was observed (data not shown).

### The combined treatment of lovastatin and gefitinib with synergistic effect on *TNF-α* expression

**Figure 2:**
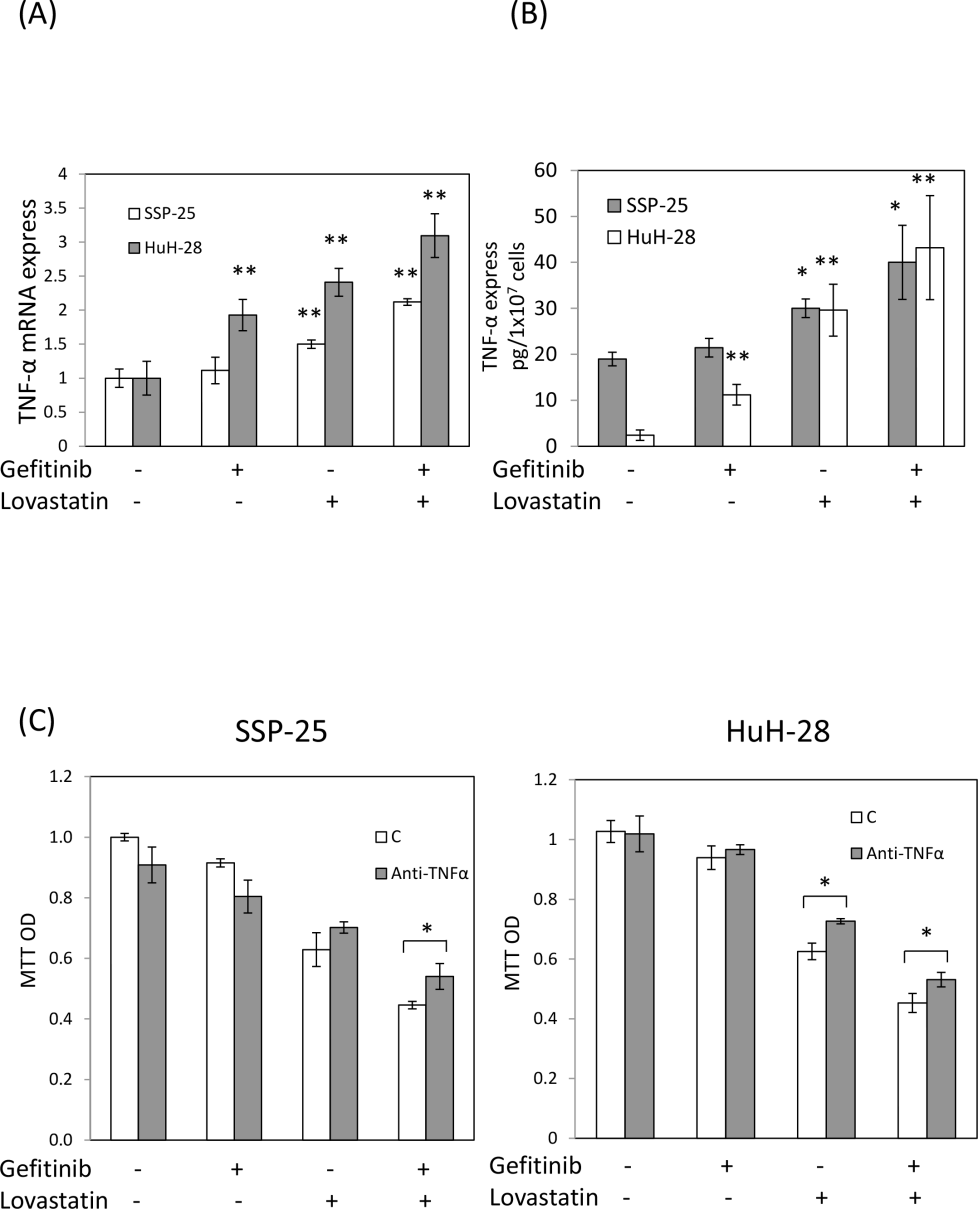
Combined treatment of lovastatin and gefitinib induced synergistic effects on the expression of *TNF*-α **A.** SSP-25 cells and HuH-28 cells (1 × 10^6^/well) were treated with lovastatin (L), gefitinib (G), or their combination (G + L) for 16 hours. Cells were harvested, and total RNA was extracted. The expression of *TNF-α mRNA* was detected using qPCR, as described in Materials and Methods. **B.** SSP-25 cells and HuH-28 cells (1 × 10^7^/well) were treated with lovastatin (L), gefitinib (G), or their combination (G + L) for 24 hours. Cells were harvested, and total protein was extracted. The expression of TNF-α expression was detected using TNF-α detect kit, as described in Materials and Methods. **C.** SSP-25 cells (left panel) or HuH-28 cells (right panel) (1 × 10^3^/well) pretreated with the anti-TNF-α antibody (0.2 μg/mL; MAB610, R&B systems) for 1 hour were treated with lovastatin (L), gefitinib (G), or their combination (G + L) for 72 hours. Cell viability was detected using the MTT assay. Student's *t* test was conducted and considered significant at *p* < 0.05 (*), 0.01 (**).

To investigate whether gefitinib enhanced lovastatin-regulated mechanisms, the expression of TNF-α was examined. The results indicated that lovastatin, but not gefitinib, induced the expression of *TNF-α mRNA* in SSP-25 cells (Fig. 2A). By contrast, both lovastatin and gefitinib induced the expression of *TNF-α mRNA* in HuH-28 cells, but lovastatin was found to be more effective (Fig. 2A). However, the combined treatment of lovastatin and gefitinib increased the expression of *TNF-α mRNA* compared with that of the signal agents in both cancer cell lines (Fig. 2A). The increased TNF-α protein was also observed in the combined treatment (Fig. 2B). To confirm the role of TNF-α in lovastatin-induced antiproliferation in both cell lines, an anti-TNF-α antibody was used to neutralize accumulated proteins in cell culture media by using the combined treatment of lovastatin and gefitinib. The results presented in Fig. 2C indicated that pretreatment with the anti-TNF-α antibody reduced lovastatin-induced an antiproliferation effect in both cell lines. This suggests that although there are different gene statuses in these two cholangiocarcinoma cell lines, gefitinib can potentiate lovastatin-induced antiproliferation through enhancing TNF-α expression.

### The combined treatment of gefitinib and lovastatin induced cell cycle arrest in HuH-28 cells through LKB1 activation

**Figure 3:**
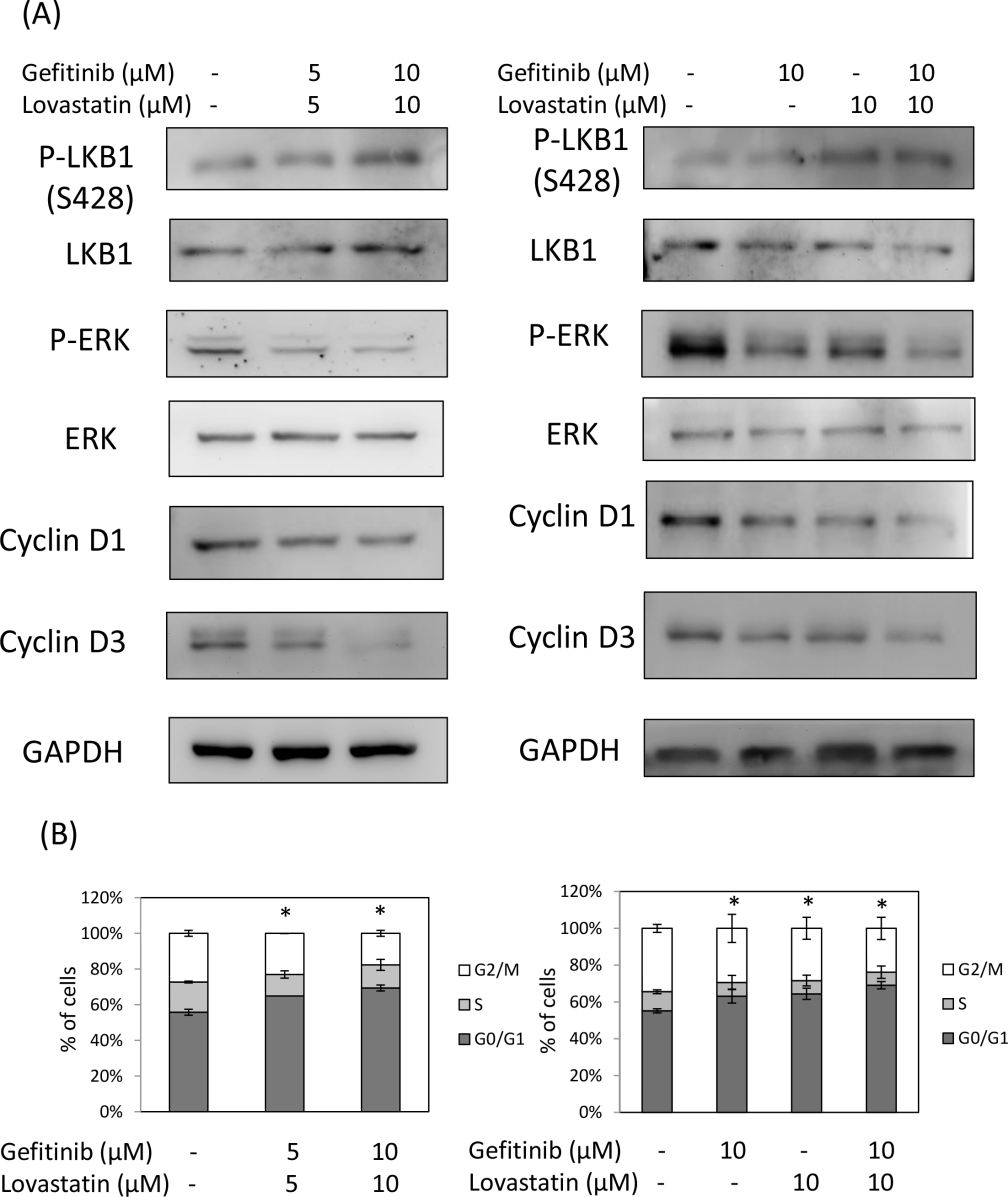

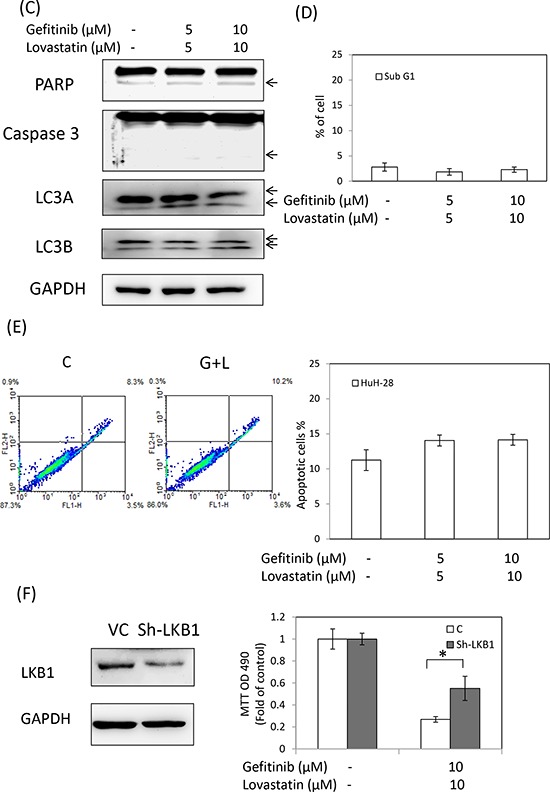
Combined treatment of lovastatin and gefitinib induced cell cycle arrest in HuH-28 cells Cells grown in six-well trays were treated with lovastatin (L) and gefitinib (G) for 24 hours. **A.** Cells were harvested, and total proteins were extracted. The cell cycle-related proteins p-LKB1, LKB1, p-ERK, ERK, cyclin D1, and cyclin D3 were detected using western blotting analyses. **B.** Cell cycle assay. Cells were harvested and fixed with ethanol. Cells were stained with RNase A/PI at 37°C for 1 hour. Flow cytometry analysis of the DNA content of the cells was performed using a FACSCalibur flow cytometer (Becton Dickinson, USA), and 10 000 events were collected and analyzed using WinMDI 2.9 software. **C.** Apoptosis and autophagy analysis. Cells were harvested, and total proteins were extracted. The cell cycle-related proteins PARP, caspase3, LC3A and LC3B were detected using western blotting analyses. **D.** Sub-G1 formation. Cells were harvested and fixed with ethanol. Cells were stained with RNase A/PI at 37°C for 1 hour. Flow cytometry analysis of the DNA content of the cells was performed using a FACSCalibur flow cytometer (Becton Dickinson, USA), and 10 000 events were collected and analyzed using WinMDI 2.9 software. **E.** Annexin V assay. Cells were harvested and stained by annexin V /Dead Cell Apoptosis Kit (Invitrogen). Flow cytometry analysis of the expression of the cells was performed using a FACSCalibur flow cytometer (Becton Dickinson, USA), and 10 000 events were collected and analyzed using WinMDI 2.9 software. **F.** HuH-28 cells were stably transfected with *shLKB1* plasmid for 72 h, and selection by puromycin. Cells were harvested, and total proteins were extracted. Total LKB1 protein was detected using western blotting analyses. HuH-28 cells stably transfected with *shLKB1* plasmid were seeded in a 96-well tray (1 × 10^3^/well) and were treated with a combination of lovastatin (L) and gefitinib (G) for 72 hours. Cell viability was detected using the MTT assay.

To further examine the mechanisms involved in gefitinib and lovastatin-induced antiproliferation in HuH-28 cell lines, apoptosis, autophagy, and the cell cycle was detected. The combined treatment of gefitinib and lovastatin increased the LKB1 activation, and downregulated park, cyclin D1, and cyclin D3 expression in a concentration-dependent manner (Fig. 3A). The results also showed that the combined treatment induced cell cycle arrest (Fig. 3B), but did not affect apoptosis or autophagy (Fig. 3C, 3D and 3E). To directly confirm the role of LKB1 in lovastatin/gefitinib treatment, the knockdown of *LKB1* expression to reduce drug-regulated antiproliferation was observed (Fig. 3F). These results suggested that the combined treatment regulated cell cycle arrest through LKB1 activation in HuH-28 cells.

### The combined treatment of lovastatin and gefitinib inhibit SSP-25 cells proliferation through apoptosis

**Figure 4:**
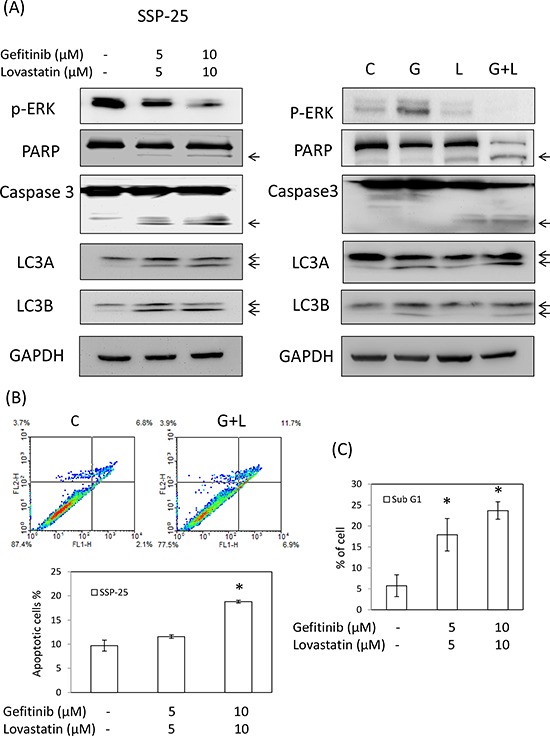

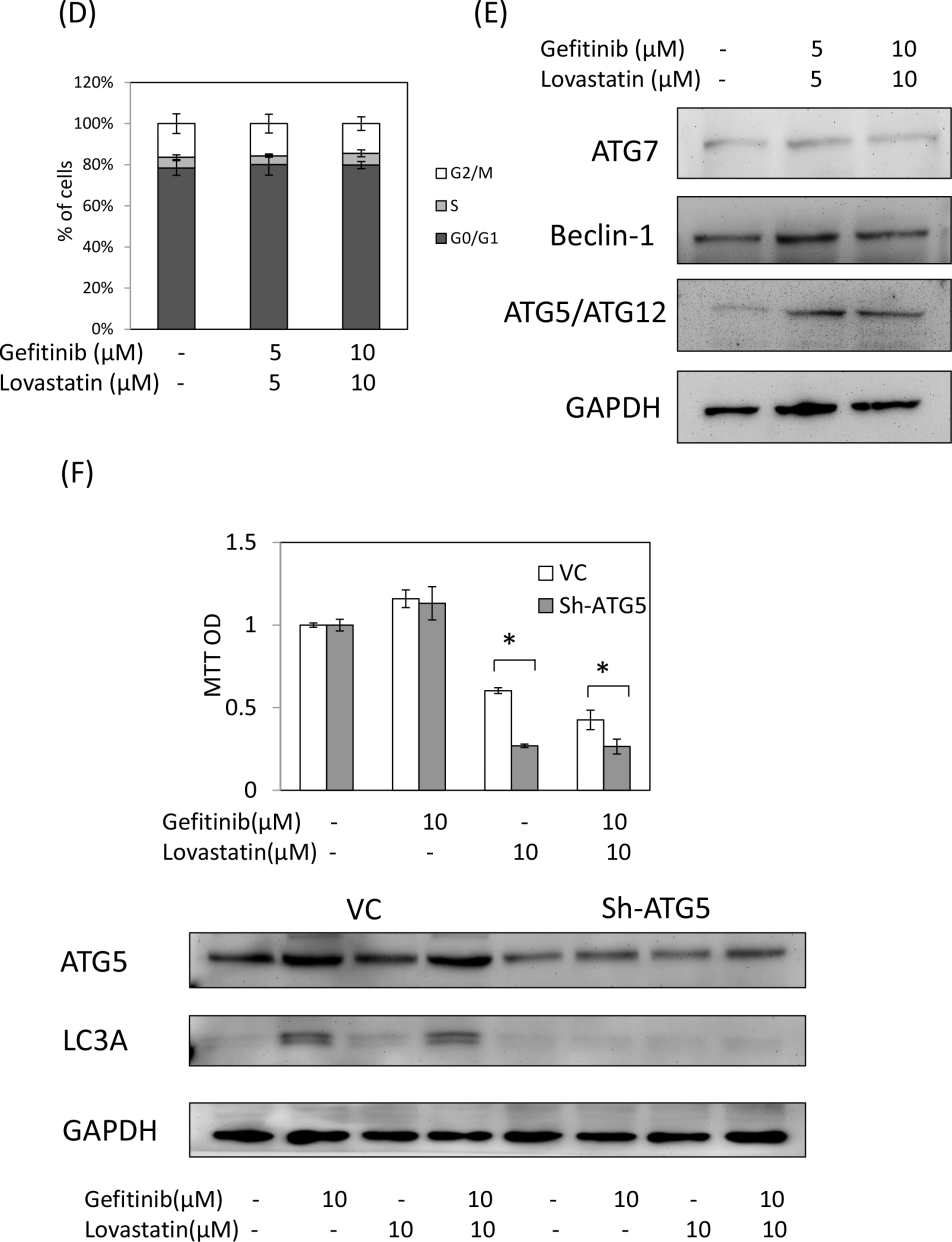

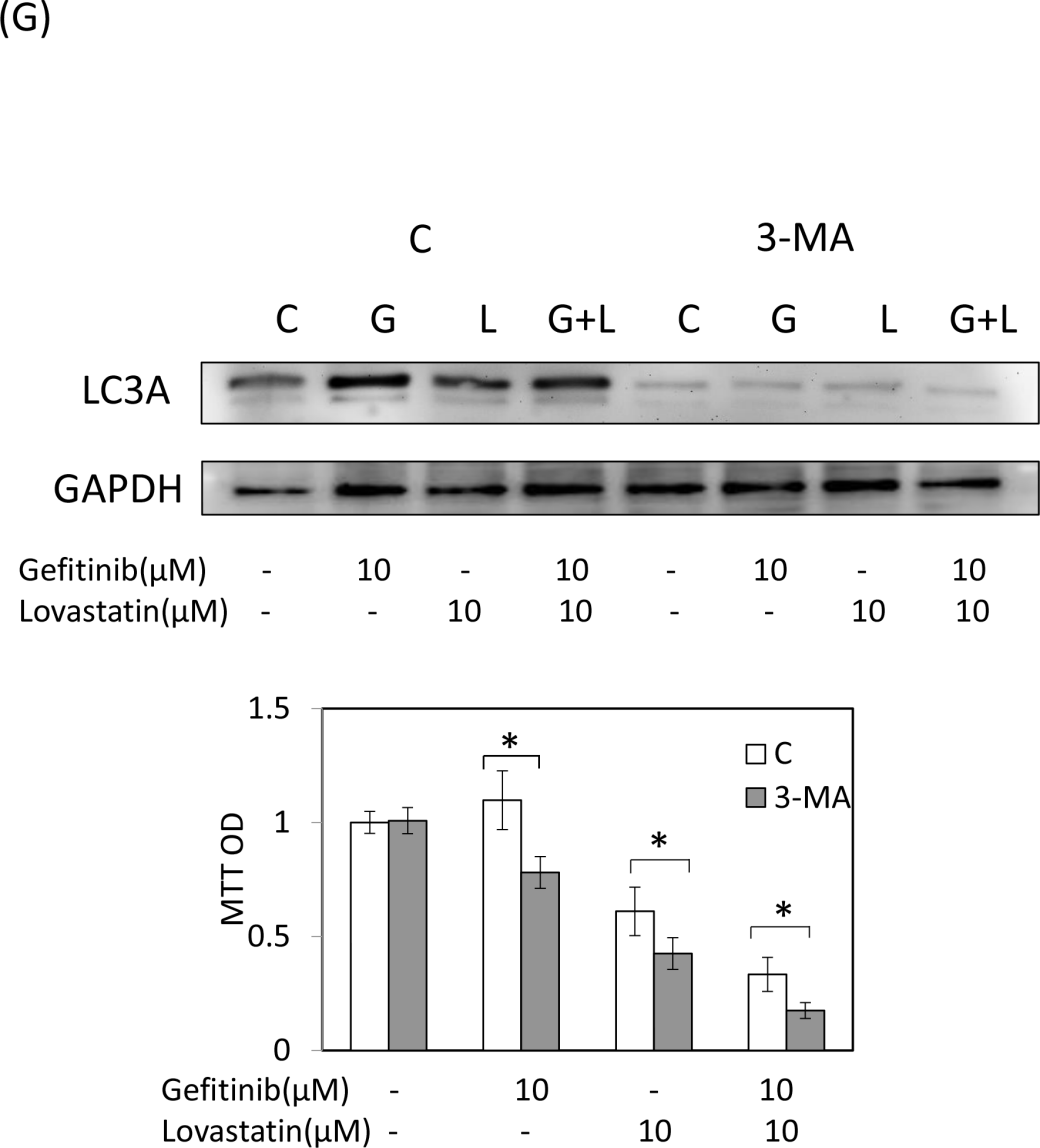
Combined treatment of gefitinib and lovastatin induced apoptosis and autophagy in SSP-25 cells The cells (1 × 10^6^/well) were seeded in a six-well tray and treated with a combination of gefitinib and lovastatin for 24 hours. Cells were harvested, and total proteins were extracted. **A.** The apoptosis markers, including the cleavage of caspase-3 and PARP and the autophagy markers LC3A and LC3B, were detected using western blotting analyses. **B.** Annexin V assay. **C.** Sub-G1 formation. **D.** The cell cycle population was detected using flow cytometry. **E.** The autophagy-related proteins, including beclin-1, ATG7, and ATG5/ATG12 complex, were detected using western blotting analyses. **F.** SSP-25 cells were stably transfected with shATG5 plasmid for 72 h, and selected by puromycin. Cells were harvested, and total proteins were extracted. Total ATG5 protein was detected using western blotting analyses. SSP-25 cells stably transfected with shATG5 plasmid were seeded in a 96-well tray (1 × 10^3^/well) and were treated with lovastatin, gefitinib, or their combination for 72 hours. Cell viability was detected using the MTT assay. Student's *t* test was conducted and considered significant at *p* < 0.05 (*). **G.** SSP-25 cells grown in 96-well trays were pre-treated with 3-MA(1 mM) for 1 hr, and treated with lovastatin (L), gefitinib (G), or their combination (G + L) for 72 hours. Cell viability was detected using MTT assay. Autophagy inhibition was detected by western blotting.

To determine whether an antiproliferation response was involved in the combined treatment of gefitinib and lovastatin in SSP-25 cells, the apoptotic markers including Annexin-V, the sub-G1, the cleavage of caspase-3, PARP, and autophagy markers LC3A and LC3B were examined in the cells treated with lovastatin, gefitinib, or their combination. The results indicated that the combined treatment of lovastatin and gefitinib induced apoptosis and autophagy (Fig. 4A, 4B and 4C) in SSP-25 cells, but did not affect the cell cycle population (Fig. 4D). The results suggest that apoptosis was induced by lovastatin while autophagy was induced by gefitinib (Fig. 4A). In order to confirm the LKB1 function in SSP-25 cell line, knockdown of LKB1 was was conducted. However, knockdown of LKB1 did not affect the gefitinib and lovastatin-induced anti-proliferation (data not show). These results demonstrated that the combined treatment of lovastatin and gefitinib-induced anti-proliferation is LKB1 independent in SSP-25. The autophagic response induced LC3A and LC3B cleavage and was activated through ATG5/ATG12 complex formation, but did not affect the ATG7 and Beclin-1 (Fig. 4E). To directly confirm the role of autophagy in the combined treatment, ATG5 was knocked down to block ATG5/ATG12 complex-induced autophagy formation and an inhibitor of authophagy, 3-MA, was also used. The results indicated that blockage of the autophagic process enhanced lovastatin-induced cytotoxicity (Fig. 4F and 4G). These results suggested that the combined treatment of gefitinib and lovastatin induced cell apoptosis and autophagy in SSP-25 cells. However, the autophagy was caused by cell survival responses.

### The combined treatment of gefitinib and lovastatin enhanced antitumor activity *in vivo*

**Figure 5:**
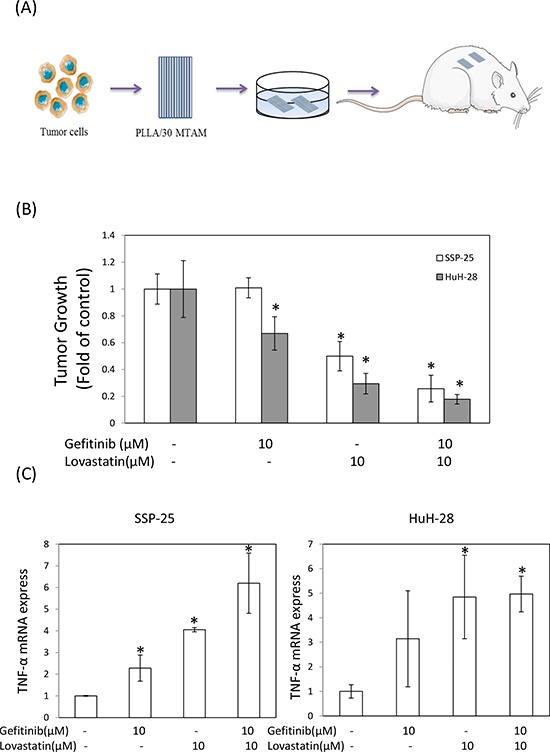
Combined treatment of lovastatin and gefitinib induced antitumor growth activity of human intrahepatic cholangiocarcinoma SSP-25 and HuH-28 cells grown in MTAMs and cultivated in mice **A.** Schematic representation of the experiment. SSP-25 or HuH-28 cells (1 × 10^5^ cells/mL) grown in MTAMs were implanted subcutaneously into mice for 72 hours, treated with drugs, and then harvested at 72 hours for **B.** the MTT assays, **C.** RT-PCR. Student's *t* test was conducted and considered significant at *p* < 0.05 (*).

**Figure 6:**
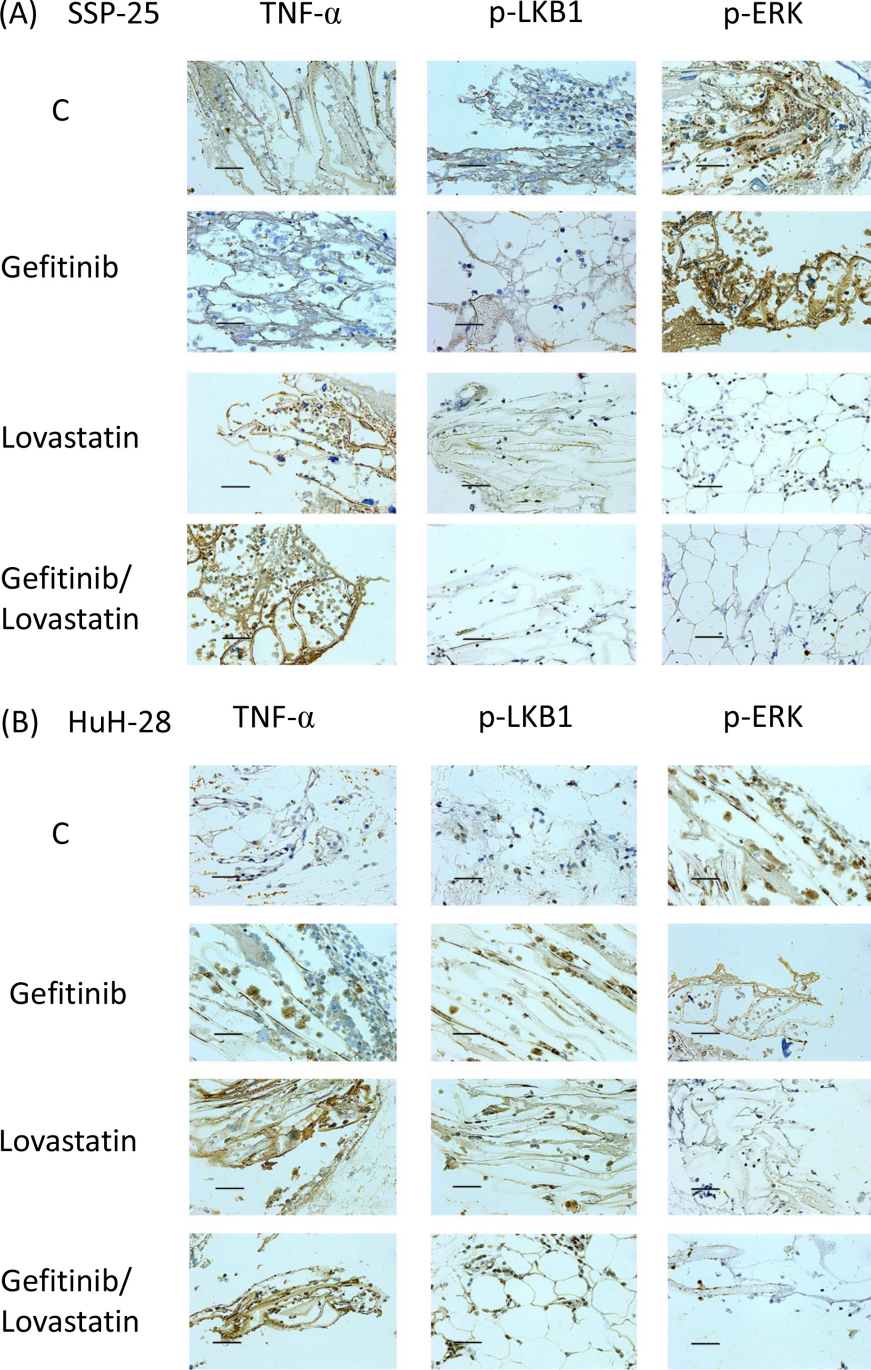

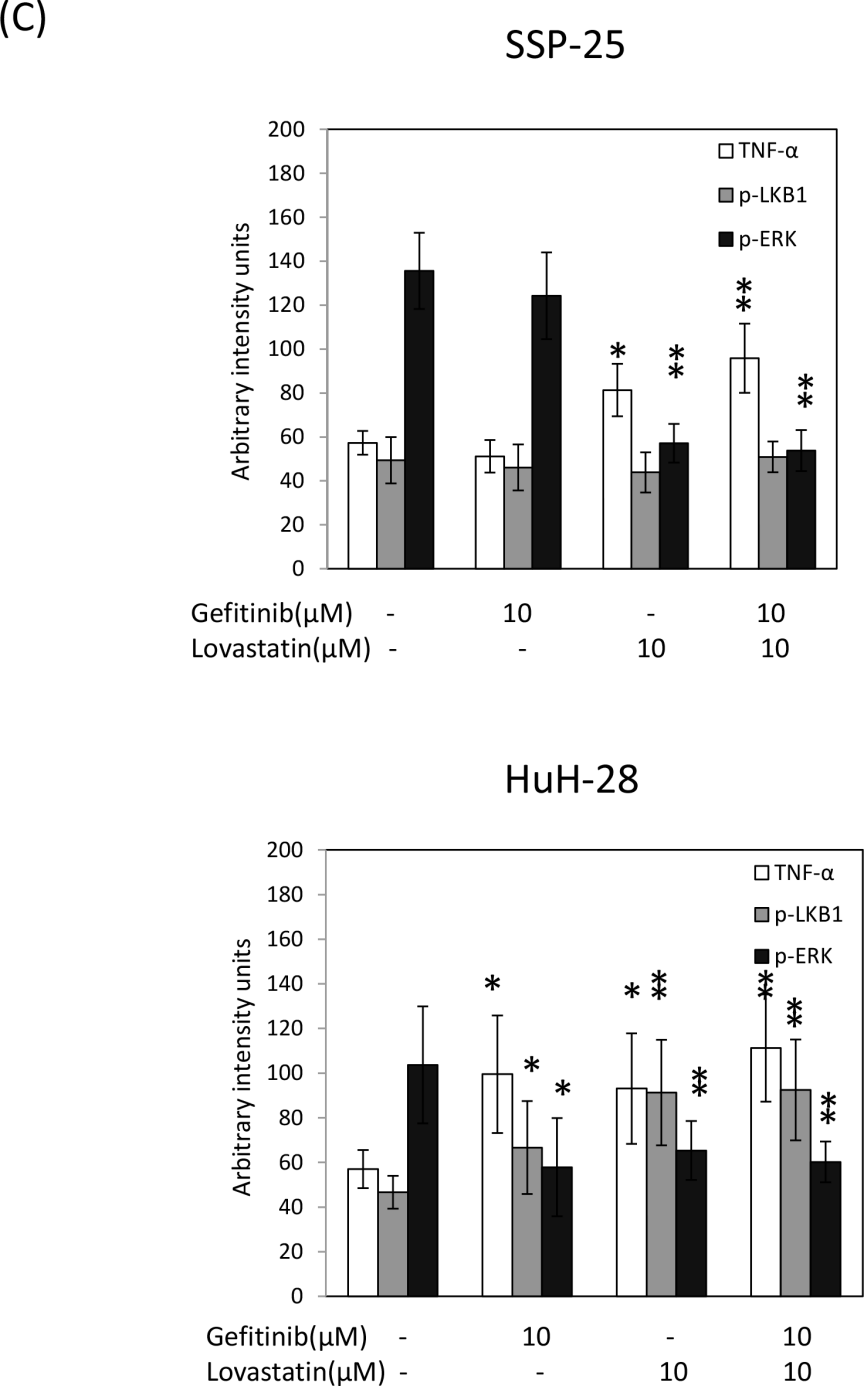
Combined treatment of lovastatin and gefitinib induced the expression of TNFα and inhibited the expression of p-ERK in SSP-25 and HuH-28 cell lines, and increased p-LKB1 in HuH-28 cell lines **A.** SSP-25 or **B.** HuH-28 cells (1 × 10^5^ cells/mL) grown in MTAMs were implanted subcutaneously into mice for 72 hours, treated with the drug, and then harvested after 72 hours; the protein expression was detected using IHC staining. The blue stain is a result of hematoxylin as a nuclear counterstain. Magnification, 400 ×. Scale bar, 20 μm. **C.** The MTAMs were imaged and calculated with the Image J software. Twenty randomly selected cells were calculated and expressed as the mean ± S.D. of intensity/cells in MTAMs. Student's *t* test was conducted and considered significant at *p* < 0.05 (*), 0.01 (**).

To evaluate the antitumor activity of the combined treatment *in vivo*, we used the hollow fiber assay in mouse models (Fig. 5A). The results of the treatment with gefitinib, lovastatin, or their combination indicated that the antitumor growth ability was consistent with that noted in studies on cell cultures (Fig. 5B). Similarly, the expression of *TNF-α mRNA* increased after treatment with these agents (Fig. 5C). These data confirmed that the combined treatment of gefitinib and lovastatin induced synergistic effects *in vitro* and *in vivo*. To further confirm the mechanism *in vivo*, we used immunohistochemistry to observe the mechanism (Fig. 6). The results indicated that the combined treatment inhibited the ERK phosphorylation and increased the expression of *TNF-α* in these two cell lines *in vivo* xenograft studies. In addition, the combined treatment also increased LKB1 phosphorylation in HuH-28 cells in xenograft.

These results suggested that although the combined treatment of gefitimb and lovastatin induced antiproliferation in SSP-25 and HuH-28 cells through different mechanisms, with the former through cell cycle arrest and the latter through apoptosis, potentiation by gefitinb of the lovastatin-induced TNF-α played a key role in the combined treatment of gefitinib-resistant human cholangiocarcinoma cells.

## DISCUSSION

The combined treatment of multiple agents on cancer has been practiced for years. The efficacy is often greater than that of the single agent. For example, atorvastatin (5 μM) enhances gefitinib cytotoxicity through concomitant inhibition of AKT and ERK activity [21]. It also interrupts K-ras/PI3K and K-ras/Raf complexes, leading to the suppression of AKT and ERK activity. Similar results were also obtained in commutant K-ras/PTEN or K-ras/PIK3CA NSCLC cells. Simvastatin ameliorates I/R-induced liver and lung tissue damaged by inhibiting the level of inflammation and the apoptotic pathways [22]. The combination of lovastatin and gefitinib effectively downregulates ras protein and suppresses the Raf, ERK1/2, AKT, and EGFR phosphorylation in gefitinib-resistant A549 and NCI-H460 human NSCLC cells [20].

Lovastatin (1–25 μM, 24 h) induces LKB1 activation in the squamous cell carcinoma (SCC) cell lines SCC9 and SCC25 [23]. LKB1 has been reported to regulate cell cycle G1 arrest through downregulated cyclin D1 and cyclin D3 [5], The results of our study indicate that the combined treatment induced LKB1 activation, sequential cyclin D1 and cyclin D3 downregulation, and cell cycle arrest in HuH-28 cells (Fig. 3). By contrast, the combined treatment of gefitinib and lovastatin induced the cell apoptosis through LKB1 indenpend pathway in SSP-25 cells (data not show). Meanwhile, increased *TNF-α* expression was observed (Fig. 2). TNF-α has been reported to induce LKB1 phosphorylation and cell apoptosis [7, 24]. These results indicated that the combined treatment induced anti-proliferation via *TNF-α* expression to lead to cell death by different mechanisms in cholangiocarcinoma cells with different LKB1 status.

EGFR tyrosine kinase inhibitors, such as gefitinib and erlotinib, activate autophagy as a cytoprotective response in human lung cancer cells [25]. Cytotoxicity induced by gefitinib or erlotinib was considerably enhanced after autophagy inhibition by the pharmacological inhibitor chloroquine and siRNAs targeting ATG5 and ATG7, the most crucial components for the formation of autophagosome [25]. In this study, the knockdown of ATG5 and the treatment with 3-MA to inhibit autophagy process enhanced the cytotoxicity induced by the combined treatment of gefitinib and lovastatin in SSP-25 cells (Fig. 4). Our data suggest that gefitinib-induced autophagy maybe crucial for cell survival.

Activation of the EGFR signal pathway reduces TNF-α-related apoptosis-inducing ligand-induced apoptosis through AKT- and XIAP-dependent mechanisms in EGFR-dependent human bladder cancer cells [26]. Studies have shown that the combined treatment of TNF-α and gefitinib is an efficient therapeutic strategy for tumors that develop resistance to gefitinib [27]. Lovastatin has been reported to increase the LPS-induced production of TNF-α [28]. In this study, lovastatin induced apoptosis or cell cycle arrest through TNF-α pathway in two gefitinib-resistant human cholangiocarcinoma cells (Figs. 3 and 4). However, the enhanced effect of the combined treatment of gefitinib and lovastatin on the lovastatin-induced expression of TNF-α was observed *in vitro* and *in vivo* (Figs. 2, 5 and 6). Additionally, pre-treatment with TNF-α antibody could reduce TNF-α-induced cytotoxicity *in vitro* (Fig. 2C). However, the concentration of TNF-α in the cell was very low (Fig. 2B), and the released TNF-α in the medium was undetected (Results not shown). These suggest that TNF-α may be undetectable in serum and thus is less impacted by TNF-α antibody in animal models. The TNF-α-induced signal transduction pathway has been shown to be a possible target of gefitinib in suppressing the intrahepatic metastasis of hepatocellular carcinoma [29]. These data demonstrate the crosstalk between the EGFR signaling pathway and TNF-α signaling pathway in cholangiocarcinoma cells; similar results were observed in human lung adenocarcinoma cells, bladder cancer cells, and hepatocellular carcinoma cells [26, 27, 29]. TNF-α treatment induces an elevated NFκB/p65 activation, leading to relatively high p21 levels and increased sensitivity to gefitinib in PC-9-ZD cells [27]. These data indicate the correlation between TNF-α and EGFR pathway. In the current study, the results show that lovastatin induces TNF-α increase and that its combination with gefitinib additionally enhances TNF-α expression [26, 27, 29].

In summary, gefitinib induced autophagy and lovastatin induced apoptosis in SSP-25 cells. By contrast, both gefitinib and lovastatin induced cell cycle arrest in HuH-28 cells. In addition, lovastatin induced the expression of TNF-α, and gefitinib enhanced lovastatin-induced antiproliferation through collaborating TNF-α expression in both cancer cell lines. Therefore, lovastatin could be used in cholangiocarcinoma cells either alone or in combination with gefitinib in human gefitinib-resistant cholangiocarcinoma cells.

## MATERIALS AND METHODS

### Cells and cell culture and drugs

The human cholangiocarcinoma SSP-25 cells and HuH-28 cells were purchased from RIKEN Bioresource Center (Ibaraki, Japan) and preserved for the study in RPMI-1640 or minimum essential medium content with 10% FBS and P/S solution (Invitrogen, Carlsbad, CA) in 5% CO_2_ incubator at 37°C. Gefitinib was purchased from Selleckchem, and lovastatin was purchased from Sigma.

### Cell viability assay

The cells (5 × 10^3^ cells/well) were seeded in 96-well plates and treated or untreated for 72 hours. Cell proliferation was determined by incubating the cells with 200 mL of a fresh medium containing 1 mg/mL 3-(4, 5-dimethylthiazol-2-yl)-2, 5-diphenyltetrazolium bromide (MTT; Sigma-Aldrich) for 4 hours at 37°C. After the MTT solution was removed, the resulting formazan crystals were dissolved completely in an ethanol/dimethyl sulfoxide mixture (1:1), and the plates were read using a microplate reader (Anthos 2010; Biochrom, Cambridge, UK) by measuring the absorbance at 490 nm. Triplicate wells were assayed for each experiment, and three independent experiments were performed. The combination index (CI) and fraction affected (Fa) of gefitinib and lovastatin for antiproliferation treatment were evaluated using the method by Chou and Talalay with CompuSyn freeware (ComboSyn Inc.; [30, 31]. Data were expressed as the mean of OD490 ± S.D.

### TNF-α expression

Human cholangiocarcinoma SSP-25 or HuH-28 cells (1 × 10^7^ cells) were seeded onto 10 cm dish. Cells were treated with gefitinib, lovastatin, or their combination for 24 hours. After the customized treatments, the cells were lyzed in cell lysis buffer (10 mM Tris, pH 7.4, 150 mM NaCl, 0.2% Triton X-100, 2 mm EDTA, 1 mM PMSF, and 1× protease inhibitor mixture), and the protein concentration was determined using the BCA assay (Thermo Scientific, Rockford, IL). The TNF-α expression was detected by TNF-α Human ELISA Kit (KHC3011, Life Technologies, Carlsbad, CA).

### ShRNA transfection

Human cholangiocarcinoma cells were seeded onto six-well tissue culture plates at 60%–80% confluence and maintained in the absence of antibiotic for 24 hours before transfection. The culture medium was removed before transfection, and the cells were washed once with PBS, then transfected with short hairpin (sh)-*RNA* for *LKB1*: CATCTACACTCAGGACTTCAC or *ATG5*: CCTGAACAGAATCATCCTTAA, or scrambled RNA (2 μg/well) purchased from the National RNAi Core Facility (Academia Sinica, Taipei, Taiwan) using lipofectamine 2000 (2 μg/well) in an Opti-MEM I medium according to the manufacturer's instructions (Invitrogen, Carlsbad, CA). After transfection, cultures were incubated at 37°C for 4 hours, and then placed in a fresh culture medium. After an additional 72 hours, the cells were used in experiments.

### Annexin V assay

SSP-25 or HuH-28 cells (2 × 10^6^ cells) in RPMI-1640 or MEM containing 10% FBS. Cells were treated with gefitinib, lovastatin, or their combination for 24 hours. Annexin V staining by using Alexa Fluor^®^ 488 annexin V /Dead Cell Apoptosis Kit (Invitrogen). The expression of the cells was performed using a FACSCalibur flow cytometer (Becton Dickinson, USA), and 10 000 events were collected and analyzed using the WinMDI 2.9 software.

### Cell cycle analysis

To measure cell cycle arrest induced by gefitinib and lovastatin, six-well tissue culture plates were seeded with SSP-25 or HuH-28 cells (2 × 10^6^ cells) in RPMI-1640 or MEM containing 10% FBS. Cells were treated with gefitinib, lovastatin, or their combination for 24 hours. Cells were washed with PBS and fixed with ethanol. Then they were stained with propidium iodide (PI; 10 μg/mL) containing RNase (1 mg/mL) at 37°C for 1 hour. Flow cytometry analysis of the DNA content of the cells was performed using a FACSCalibur flow cytometer (Becton Dickinson, USA), and 10 000 events were collected and analyzed using the WinMDI 2.9 software.

### Western blotting

After customized treatments, the cells were lyzed in cell lysis buffer (10 mM Tris, pH 7.4, 150 mM NaCl, 0.2% Triton X-100, 2 mm EDTA, 1 mM PMSF, and 1× protease inhibitor mixture), and the protein concentration was determined using the BCA assay (Thermo Scientific, Rockford, IL). Cell lysates were separated on SDS-PAGE and transferred to nitrocellulose membranes; they were probed with an antibody against PARP-1(GTX100573), ERK1(113094), and GAPDH(100118) purchased from Genetex Inc. (San Antonio, TX), and LKB1 (ab15095), p-LKB1(Ser428)(ab63473) purchase from Abcam(Cambridge, UK) and p-ERK (Thr202/Tyr204)(4377), LC3A(4599), LC3B(3868), Caspase3(9665), ATG5(8540), ATG7(8558), beclin1(4597), cyclin D1(2926), and cyclin D3(2936) were purchased from Cell Signaling Technology (Beverly, MA). Secondary antibodies were either goat anti-rabbit IgG or rabbit anti-mouse IgG (1:3000), depending on the origin of the primary antibody. Immunoreactive proteins were detected using the BioSpectrum 810 Imaging System (UVP).

### Quantitative real-time PCR

Total RNA was extracted using the RNeasy Micro Kit (Qiagen, Venlo, the Netherlands), and cDNA synthesis was performed using the RevertAid™ H Minus First Strand cDNA Synthesis Kit (Thermo Scientific). Quantitative PCR was conducted with 5 μL of DNA combined with 10 μL of IQ SYBR Green supermix (Bio-Rad, Hercules, CA, USA), 0.3 μL each of 20 μM forward and reverse primers, and 4.7 μL DNase/RNase free water. The sequences for the amplified primers are as follows: TNFα forward: 5′-GCCCATGTTGTAGCAAACCC-3′ and reverse 5′-TATCTCT CAGCTCCACGCCA-3′, GAPDH forward: 5′-AGGGCTGCTTTTAACTCTGGT-3′ and reverse 5′-CCCCACTTGATTTTGGAGGGA-3′. The reactions were performed in a CFX Connect™ Real-Time PCR Detection System (Bio-Rad).

### Preparation of poly-L-lactic acid (PLLA) microtube array membranes (MTAMs)

Materials used were PLLA (BioTechOne, Taiwan), polyethylene glycol/polyethylene oxide (PEG/PEO; Sigma-Aldrich), *N, N*-dimethyl formamide (DMF; Tedia, USA), and dichloromethane (DCM; Mallinckrodt, USA). To prepare the electrospinning dopes, PLLA was dissolved in mixed DCM/DMF (8/2) solvents at room temperature to obtain a 10% solution. PEG was added to the PLLA solution to obtain PEG/PLLA (30/70) solutions, as described previously. [32–34] An electrostatics charger (ChargeMaster, Simco-Ion, Alameda, CA, USA) or a high voltage power supply unit (You-Shang Co., Fongshan city, Taiwan) was used as the source of electrostatics. Typically, electrospinning was performed by delivering the PLLA (shell) and PEG (core) solutions through a house-made co-axial spinneret with a syringe pump (KDS-100, KD Scientific, Holliston, MA, USA) at the rate of 4–9 mL/h, with 5–7 kV of applied voltage and 3–5 cm of distance to a rotating drum collector. All electrospinning procedures were performed in a chamber at a relative humidity of 50% ± 5% and a temperature of 25°C ± 1°C. PLLA MTAMs were obtained by washing to remove the core component of PEG, followed by drying.

### Xenograft study

SSP-25 cells have been reported to exhibit poor tumorigenesis [35]. A hollow fiber model was used to replace the conventional xenograft in this study [4]. A novel hollow fiber substrate-MTAMs were synthesized and provided by Dr. Chen Chien-Chung (TMU, Taipei, Taiwan) [36]. Twenty male ICR outbred mice (10 wk old) were obtained from Charles River Laboratories (Wilmington, US) and purchased from the BioLASCO (Yi-Lan, Taiwan). Animal breeding was performed according to the guidelines of the Institutional Animal Care and Use Committee, which are based on the guidelines of the Association for Assessment and Accreditation of Laboratory Animal Care including facility. Mice were housed in the TMU Laboratory Animal Center (Taipei, Taiwan) in a conventional environment at constant temperature (20°C ± 3°C) and humidity (50% ± 20%). The animals had free access to autoclaved dry granule food and water during the study period. Each animal was seeded with two MTAMs with different cells grown (1 × 10^5^) on the backside. After 3 days of cell proliferation, the animals were treated intravenously with gefitinib (10 mg/kg/d), lovastatin (10 mg/kg/12 h), and their combination (as shown in Fig. 5A). The MTAMs were harvested 72 hours after injection. A general clinical observation (e.g., in life numbers, body weight, food consumption) was made twice daily by a veterinarian.

### Immunohistochemistry

MTAMs were separated from mice and were formalin-fixed and paraffin-embedded. Sections (3 μm thick) were placed onto adhesive-coated glass slides, dewaxed, rehydrated with PBS, and followed by incubation with the primary antibody TNF-α (SC-8301)(1:200 dilution; Santa Cruz Biotechnology, CA, USA); p-LKB1(Ser428)(ab63473) (1:200 dilution; Abcam, Cambridge, UK); p-ERK (Thr202/Tyr204)(4377) (1:200 dilution; Cell Signaling Technology, Beverly, MA). Immunocomplexes were detected using the Novolink Polymer Detection System (Leica Biosystems Newcastle Ltd., UK), and the slides were then counterstained with hematoxylin. The MTAMs were imaged and twenty randomly selected cells calculated by Image J software [3].

### Data analysis and statistics

All the quantitative data were repeated thrice (with sample number ≥ 3), and the mean values were plotted with standard deviations. The statistical significance was calculated using Student's *t* test.
